# Mental health of UK firefighters

**DOI:** 10.1038/s41598-022-24834-x

**Published:** 2023-01-10

**Authors:** Taylor A. M. Wolffe, Andrew Robinson, Anna Clinton, Louis Turrell, Anna A. Stec

**Affiliations:** 1 Centre for Fire and Hazards Sciences, University of Central Lancashire, Preston, Lancashire PR1 2HE UK; 2 Royal Preston Hospital, Lancashire Teaching Hospitals NHS Foundation Trust, Preston, Lancashire, PR2 9HT, UK

**Keywords:** Cancer prevention, Environmental sciences, Risk factors

## Abstract

Exposure to trauma, high-stress situations, and disrupted sleep are well known risk factors affecting firefighters’ mental health. Little is known about the association between firefighters’ exposure to fire contaminants and mental health disorders. The UK Firefighter Contamination Survey assessed firefighters’ health and capacity for occupational exposure to contaminants. Participants were invited to anonymously complete its 64 questions online. Logistic regression analyses assessed the associations between self-reported mental health disorders and proxies of contaminant exposure. Results found that firefighters who notice soot in their nose/throat for more than a day after attending fires (Odds Ratio (OR) = 1.8, 1.4–2.4), and those who remain in their personal protective equipment (PPE) for over 4 h after fires (OR = 1.9, 1.2–3.1), were nearly twice as likely to report mental health disorders. Significantly increased odds ratios for all three outcomes of interest (anxiety, depression and/or any mental health disorders) were also found among firefighters who take PPE home to clean. Sleeping problems were reported by 61% of firefighters. These firefighters were 4.2 times more likely to report any mental health disorder (OR = 4.2, 3.7–4.9), 2.9 times more likely to report anxiety (OR = 2.9, 2.4–3.5) and 2.3 times more likely to report depression (OR = 2.3, 1.9–2.8) when compared to firefighters who did not report sleep issues. Effective decontamination measures within UK Fire and Rescue Services, together with firefighters’ wellness, may play a crucial role in protecting firefighters’ mental health.

## Introduction

Mental health disorders can be caused by a combination of psychological, environmental, biological, and chemical factors. To date, research on firefighters’ mental health has mainly focused on psychological factors such as direct exposure to trauma^1^ or occupational stress^2^, finding firefighters to have an increased risk of suicide^3^, depression^1,2^, and post-traumatic stress disorder (PTSD)^4^. Studies have also investigated the effects of other occupational exposures, e.g. abrupt fire incident call-outs, disrupted sleep^5^, as well as physical and/or emotional fatigue on firefighters’ mental health. However, little is known about the relationship between firefighters’ exposure to fire effluent and mental health.

Fire smoke is a heterogenous cocktail of chemicals with varying toxicities and modes of action^6^. A growing body of research has identified a number of different fire effluents contaminating fire stations and firefighters’ PPE. Elevated levels of polybrominated diphenyl ethers (PBDEs)^7,8^, polychlorinated and polybrominated dibenzo-p-dioxins and dibenzofurans (PCDD/Fs and PBDD/Fs)^9^ or halogenated gas phase flame retardants^7^ have also been reported in firefighters’ blood and urine^10^– suggesting several routes for chronic exposure^11–16^.

Several components of fire smoke have also been associated with mental health disorders. For example, the U.S. Environmental Protection Agency and the Agency for Toxic Substances and Disease Registry, found exposure to neurotoxic chemicals such as mercury and lead, or endocrine disruptors such as polychlorinated biphenyls, PBDEs or poly- and perfluoroalkyl substances (PFAS), can lead to hormone imbalances and/or neuroendocrine dysfunction, leading to conditions such as depression and anxiety^17^. Additionally, suffering life-altering, painful and/or chronic physical health conditions (e.g. cancer or other long-term diseases) from exposures to contaminants may also negatively impact firefighters' mental health.

The UK Firefighter Contamination Survey has uncovered several practices which would increase firefighters’ risk of contaminant exposure and/or promote travel of contaminants back to workplaces/homes^18^. This manuscript explores one of potentially many health implications of such increased exposure, associating proxies of contamination with self-reported mental health disorders among UK firefighters (see also Wolffe et al.^18^). By assessing mental health risks for a broad range of contaminant control practices the survey provides an interim means of quickly identifying areas in which UK Fire and Rescue Services can optimally target resources for improving firefighters’ occupational health.

## Methods

### Survey design

The methods used to conduct the survey and analyse its results are detailed in Wolffe et al.^18^ and are summarised again in Supplemental File S2. Ethical approval for the survey was granted by the University of Central Lancashire Ethics Committee and all analyses were conducted in accordance with relevant guidelines and regulations.

Briefly, all currently serving (i.e. excluding retired) UK firefighters were eligible to take part in the survey and were recruited to participate via email through the Fire Brigades Union (whose members make up approximately 75% of the UK’s total firefighting workforce^18,19^). Firefighters were invited to anonymously complete the survey online. Informed consent was obtained from all participants.

The survey, which took approximately 20 min to complete, consisted of 64 questions covering six key topics (Tables S1, S2, S3, S4, S5 and S6): demographics, PPE, workplace contamination, personal contamination, attitudes/awareness and training, and health (Supplemental File S1). Branching logic was used to route participants through the survey based on answers to previous questions.

### Mental health conditions

**Figure 1:**
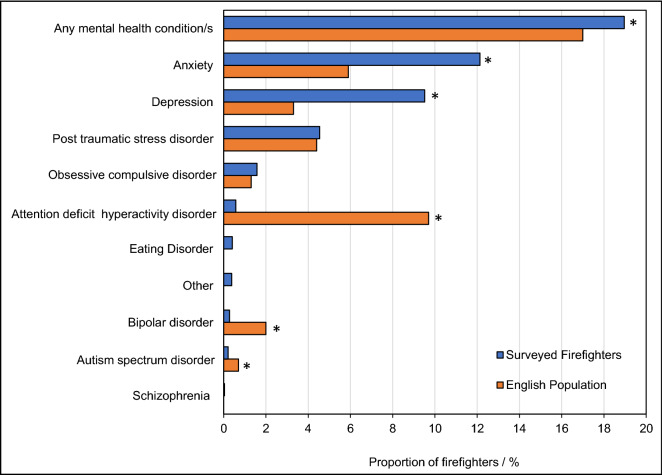
**Firefighters’ Mental Health Conditions.** The proportion of total surveyed firefighters with any, or specific, mental health disorders (blue). The prevalence of mental health disorders in the general English population^39^ is displayed for comparison (orange). “Any mental health condition”, “anxiety” and “depression” among surveyed firefighters is compared to “any common mental disorder”, “generalised anxiety disorder” and “depressive episode” in the general English population, respectively. * indicates a *p*-value of < 0.05 for differences in proportions between firefighters and English population.

Firefighters were able to choose multiple specific conditions from a list, as presented in Fig. 1 and Supplemental File S1. Analyses conducted in this manuscript specifically assess *any* mental health condition (i.e. those reporting at least one of the listed mental health conditions), anxiety, or depression.

### Analysis

A complete case analysis was conducted for all firefighters who answered the questions about mental health and potential contaminant exposure.

Multiple logistic regression analyses were used to assess the association between mental health conditions and potential for exposure to fire contaminants, adjusting for plausible non-contaminant risk factors and confounders assessed in the survey (see Supplemental File S2) i.e. age, length of service, sleeping problems^20^, excessive drinking^21,22^, smoking^23^, infrequently exercising^24^, having a managerial role (as a measure of work-related stress^25^), attending fires on a more frequent basis (regular exposure to trauma^26^), having another physical health condition (i.e. diabetes^27,28^, blood pressure problems^29,30^, cancer^31,32^, or fertility issues^33–35^), and having another mental health condition^36^ (for analyses of depression and anxiety).

Analyses were conducted using the statsmodels module for Python 3^37^, and Statistical Package for Social Sciences (SPSS) version 28.0.1.1. Note that firefighters reporting only attention deficit hyperactivity disorder (ADHD) or only autism spectrum disorder (ASD) were excluded from logistic regression analyses as these conditions represent developmental disorders, first appearing in childhood, and most commonly researched with regard to early life exposures to contaminants, rather than the later life exposures firefighters receive as adults during their careers.

Odds ratios (OR) with 95% confidence intervals are presented as a means of assessing the statistical significance of differences in the prevalence of mental health conditions between various demographic groups, or between groups at (potentially) greater versus lower risk of contaminant exposure.

## Results and analysis

A total of 10, 649 firefighters responded to the questionnaire, representing approximately 24% of the UK’s total firefighter workforce (Wolffe et al.^18^). Participant demographics were analysed in Wolffe et al. ^18^, and were not found to significantly differ from the English firefighter population^38^ with respect to sex (*p* > 0.05), but appeared to under-represent younger age categories, retained firefighters, and those belonging to an ethnic minority (*p* < 0.05)^18^.

Around 19% (n = 2019) of firefighters self-reported at least one mental health condition. Figure 1 displays the range and frequency of mental health disorders selected by firefighters, which is then compared to the prevalence of those conditions in the general English population (where possible^3^). Note that firefighters were able to select more than one mental health condition.

Self-reports of anxiety were most prevalent among surveyed firefighters (approximately 12%), followed by depression (around 10%) and post-traumatic stress disorder (PTSD) (around 5%), as presented in Fig. 1. The prevalence of self-reported anxiety and depression among surveyed firefighters was also significantly higher than that of the English population (Fig. 1, difference in proportions test *p* < 0.05).

Less than 2% of surveyed firefighters reported obsessive compulsive disorder (OCD) (1.6%), attention deficit hyperactivity disorder (ADHD) (0.6%), eating disorders (0.4%), bipolar disorder (0.3%), autism spectrum disorder (ASD) (0.2%), or schizophrenia (0.04%). The majority of firefighters who selected “other” (0.4%) listed “stress” associated with their work (40% of firefighters answering “other”, or 0.2% of total surveyed firefighters).

**Figure 2:**
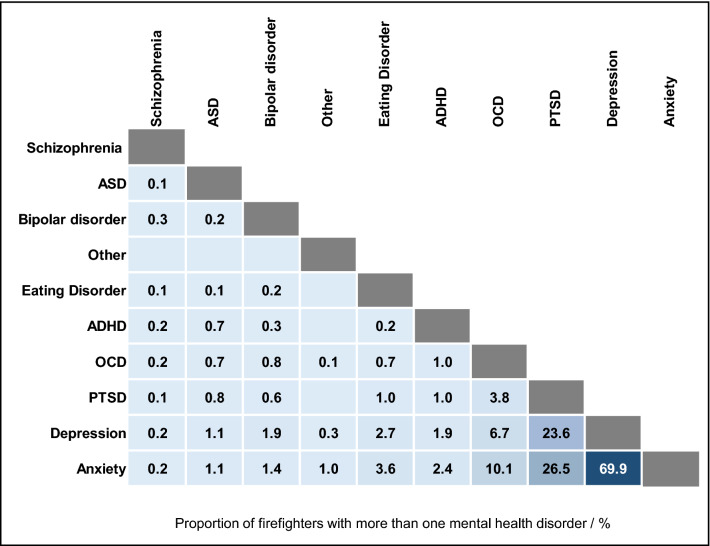
**Firefighters’ Co-occurring Mental Health Conditions.** Proportion of firefighters with more than one mental health condition (n = 878), who reported the co-occurrence of specific mental health conditions. Blank cells indicate that mental health conditions did not co-occur among surveyed firefighters.

Out of the 19% of surveyed firefighters suffering any mental health condition (n = 2019), around 44% reported having more than one condition (n = 878). The frequency with which mental health conditions co-occurred is displayed in Fig. 2. Anxiety and depression co-occurred most frequently.

From the logistic regression models (Supplemental File S2), having a co-occurring mental health condition had the most sizeable impact on reporting depression or anxiety. Firefighters with at least one co-occurring condition were nearly 16 times more likely to suffer depression (OR = 15.6, 13.3–18.4) and around 13 times more likely to suffer anxiety (OR = 13.3, 11.5–15.5) when compared to firefighters with no co-occurring mental health conditions (i.e. who only had depression, or only anxiety).

### Demographics

**Figure 3:**
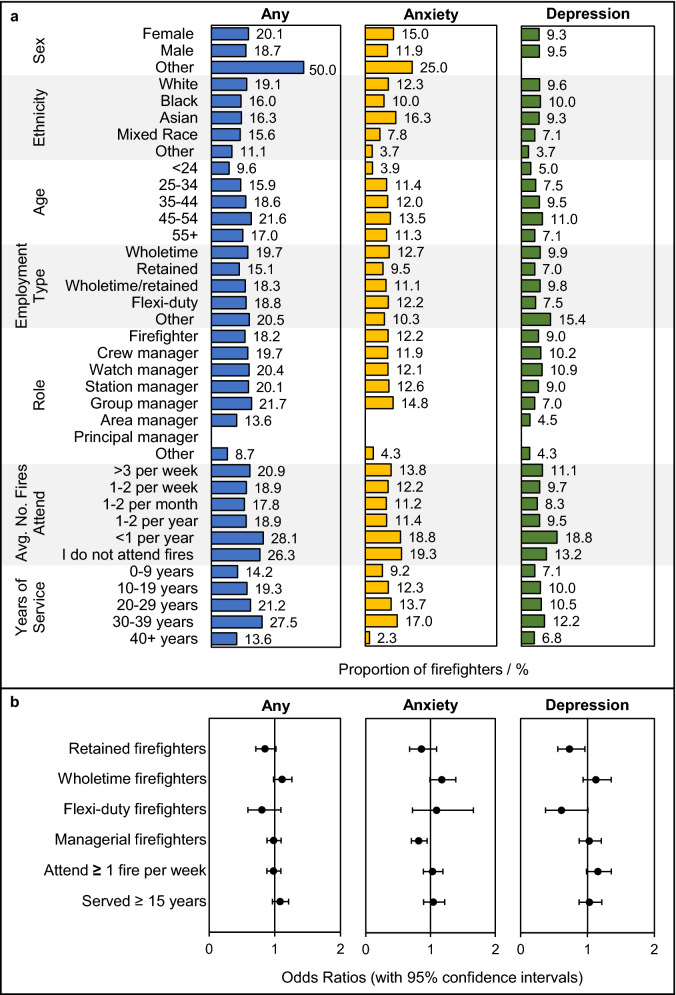
**Firefighters’ Mental Health Conditions** **by Demographics**. (**a**) The proportion of firefighters with any mental health condition, anxiety, or depression in each demographic category. (**b**) Odds ratios (with 95% confidence intervals) for specific demographic groups having any mental health condition, anxiety, or depression. Note that the proportions of surveyed firefighters in each demographic category are presented in Wolffe et al., ^18^. The results of underpowered demographic groups (e.g. sex = “Other” for which there was a total of 4 firefighters) should be interpreted cautiously. Odds ratios were adjusted for various mental health risk factors (Supplementary File S2).

Similar proportions of firefighters in each role, except for the most senior roles (such as area or principal manager), were found to suffer from any mental health condition, anxiety, or depression, Fig. 3. The proportion of surveyed firefighters with any mental health condition, anxiety, or depression generally increased with length of service, Fig. 3. Firefighters who had served longer (i.e. ≥ 15 years) were slightly more likely to report suffering any mental health condition compared to firefighters who had served less time (OR = 1.1, 1.0–1.3), although no significant differences were observed for anxiety (OR = 1.0, 0.9–1.2) or depression (OR = 1.0, 0.9–1.2).

Only wholetime firefighters, when compared to firefighters on all other contract types combined, were more (but not significantly more) likely to report any mental health condition (OR = 1.1, 1.0–1.3), and anxiety (OR = 1.2, 1.0–1.4).

Firefighters who attended fires on at least a weekly basis were more likely to report depression compared to firefighters who attended fires less frequently (OR = 1.2, 1.0–1.4). However, no significant differences between these groups were noted for any mental health condition (OR = 1.0, 0.9–1.1), or anxiety (OR = 1.0, 0.9–1.2).

### Health and lifestyle

Firefighters were also asked questions related to their health and lifestyle, which could possibly be linked with mental health conditions such as anxiety, or depression (Supplemental File S1, Wolffe et al.^18^). These questions were subsequently included in logistic regression models (Supplemental File S2) and are explored further below.

#### Sleeping problems

**Figure 4:**
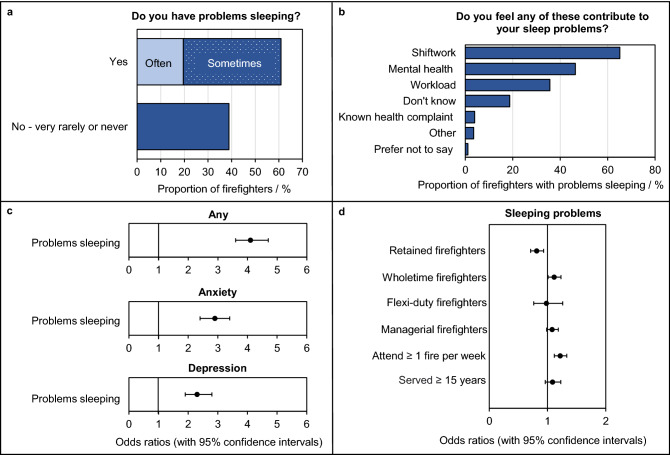
**Firefighters’ Sleeping Problems.** (**a**) The proportion of total surveyed firefighters who indicated whether they had sleeping problems. (**b**) The proportion of firefighters with sleeping problems listing the reasons for their sleep disturbances. Note that firefighters were able to select more than one reason. (**c**) Adjusted odds ratios (with 95% confidence intervals) for firefighters’ mental health conditions due to sleeping problems. (**d**) Adjusted odds ratios (with 95% confidence intervals) for firefighters sleeping problems due to demographic variables. Note that odds ratios presented in (**c**) and (**d**) were adjusted for a variety of disturbed sleep risk factors (Supplementary File S2).

Around 61% of all surveyed firefighters (n = 6490) said that they have sleeping problems, Fig. 4a. Those who reported sleeping problems were 4.2 times more likely to report any mental health condition (OR = 4.2, 3.7–4.9), 2.9 times more likely to report anxiety (OR = 2.9, 2.4–3.5) and 2.3 times more likely to report depression (OR = 2.3, 1.9–2.8) compared to firefighters who did not report it, Fig. 4c.

Firefighters were also asked to provide more details about their sleeping problems. They were able to select multiple answers, listed in Supplemental File S1. Over 65% of firefighters (out of 6490, n = 4228) indicated that shift work was a major sleep disturbance (Fig. 4b). Mental health (46%, n = 3010) and workload (36%, n = 2313) were also frequently selected. Home, personal or family-life related reasons (e.g. disturbed by children or split/divorce etc.) were the most common disturbances listed by firefighters selecting “other”.

Multiple logistic regression analyses (Supplemental File S2) revealed a slightly increased likelihood of reporting sleeping problems for managerial firefighters (OR = 1.1, 1.0–1.2), wholetime firefighters (OR = 1.1, 1.0–1.2), firefighters attending fires on at least a weekly basis (OR = 1.2, 1.1–1.3), and firefighters with 15 + years of service (OR = 1.1, 0.9–1.2), Fig. 4d. However, this increase was only significant for firefighters attending incidents on a weekly basis. Retained firefighters were slightly, but significantly, less likely to report sleeping problems when compared to firefighters employed on all other contract types combined (OR = 0.9, 0.7–1.0).

#### Other health and lifestyle variables

Several other health and lifestyle variables were also found to be significantly associated with firefighters’ mental health, and are presented in Table 1.

**Table 1:** Firefighters’ other health and lifestyle factors found to be significantly associated with their mental health.

	Lifestyle variables	Odds ratio (OR) with 95% confidence intervals
Any mental health condition	Blood pressure problems	1.5 (1.3–1.8)
	Cancer diagnosis	1.5 (1.1–1.9)
	Fertility problems	1.4 (1.2–1.7)
	Excessive drinking	1.2 (1.0–1.4)
	Smoking	1.3 (1.1–1.5)
Anxiety	Blood pressure problems	1.4 (1.1–1.7)
	Fertility problems	1.3 (1.0–1.7)
Depression	Exercising infrequently	1.4 (1.1–1.7)
Sleeping problems	Blood pressure problems	1.5 (1.2–1.7)
	Excessive drinking	(1.1–1.4)
	Smoking	1.2 (1.1–1.4)
	Fertility problems	1.3 (1.1–1.5)

Odds ratios were adjusted for several mental health risk factors (e.g. sleeping problems) and/or sleep disturbances (e.g. age), detailed in Supplemental File S2, Tables S2, S3 and S4. Excessive drinking refers to the consumption of more than 15 units of alcohol per week (one unit equals 10 mL of pure alcohol).

Firefighters with a cancer diagnosis were significantly more likely to report any mental health condition (OR = 1.5, 1.1–1.9), but were not found to be significantly more likely to report anxiety (OR = 1.2, 0.9–1.7), depression (OR = 0.9, 0.6–1.3) or sleeping problems (OR = 1.0, 0.8–1.3).

Aside from problems sleeping and having a co-occurring mental health condition, the only health/lifestyle variables found to be significantly associated with depression was infrequent exercising (OR = 1.4, 1.2–1.7). Excessive drinking, smoking, and problems with blood pressure were found to be significantly associated with both any mental health condition and sleeping problems (Table 1).

Fertility problems were significantly associated with any mental health condition (OR = 1.4, 1.1–1.9), anxiety (OR = 1.3, 1.0–1.7) and sleeping problems (OR = 1.3, 1.1–1.5).

### Exposure to fire contaminants during/after fire incidents

**Figure 5:**
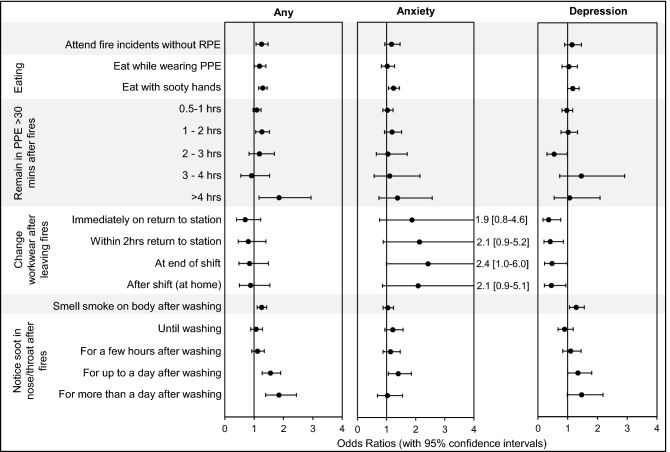
**Firefighters’ Exposure to Contaminants During/After Fires and Mental Health Odds Ratios.** Odds ratios (with 95% Confidence Intervals) were adjusted for various mental health risk factors (Supplementary File S2).

Significantly increased odds ratios for any mental health condition were found for firefighters who noticed soot in their nose/throat for more than a day after attending a fire (OR = 1.8, 1.4–2.4), and for those that remained in PPE for more than 4 h after a fire (OR = 1.9, 1.2–3.1). Results are shown in Fig. 5. Firefighters were also significantly more likely to report any mental health condition if they reported still noticing the smell of fire smoke on the body after washing (OR = 1.3, 1.1–1.5), or eating with sooty hands (OR = 1.3, 1.1–1.4).

Similar results were found for anxiety. Significantly increased odds ratios were found for firefighters who eat with sooty hands (OR = 1.2, 1.1–1.4 for anxiety), and who noticed soot in the nose/throat for up to a day after washing (OR = 1.4, 1.0–1.8 for anxiety).

Those noticing the smell of fire smoke on the body after washing were significantly more likely to report depression (OR = 1.3, 1.0–1.5).

### Fire contaminants on firefighters’ PPE and at their workplace

**Figure 6:**
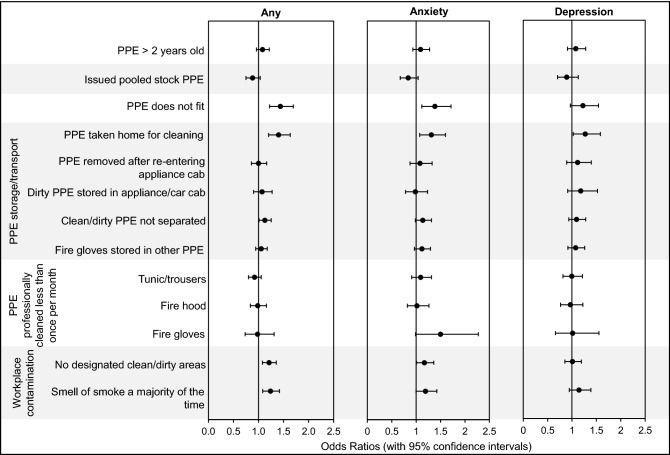
**Firefighters’ PPE/Workplace Contamination and Mental Health Odds Ratios.** Odds ratios (with 95% confidence intervals) were adjusted for various mental health risk factors (Supplementary File S2).

Firefighters with ill-fitting PPE were significantly more likely to report any mental health disorder (OR = 1.4, 1.2–1.7), and anxiety (OR = 1.4, 1.1–1.7), presented in Fig. 6. Significantly increased odds ratios were also found for firefighters taking PPE home (OR = 1.4, 1.2–1.6 for any mental health condition, OR = 1.3, 1.1–1.6 for anxiety, and OR = 1.3, 1.0–1.6 for depression).

Additionally, significantly increased odds ratios were found, for those firefighters failing to store clean and dirty PPE separately (OR = 1.1, 1.0–1.3 for any mental health condition (Fig. 6)).

Infrequently sending fire gloves for professional decontamination was also associated with an increased anxiety odds ratio (OR = 1.5, 1.0–2.3).

Firefighters who worked in stations with no designated clean and dirty areas were also more likely to report any mental health condition (OR = 1.2, 1.1–0.4), and anxiety (OR = 1.2, 1.0–1.4), as were firefighters working in stations which smell of fire (OR = 1.2, 1.1–1.4 for any mental health condition and OR = 1.2, 1.0–1.4 for anxiety), Fig. 6.

### Culture, fire contaminant awareness and training and culture

**Figure 7:**
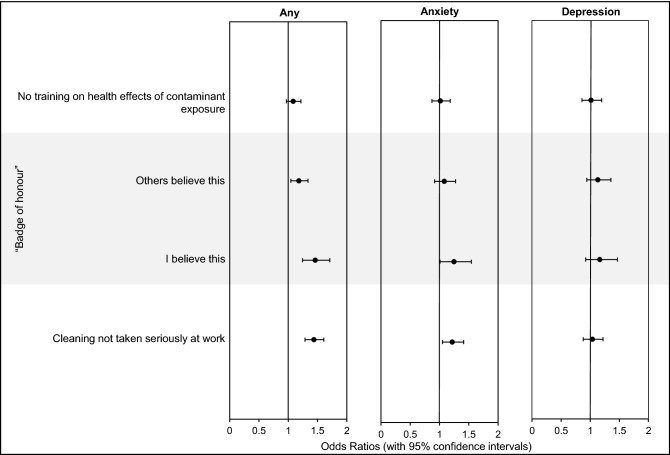
**Firefighters Training/Culture and Mental Health Odds Ratios.** Odds ratios (with 95% confidence intervals) were adjusted for various mental health risk factors (Supplementary File S2).

Firefighters who personally believe that contaminated PPE should be celebrated as a “badge of honour” (explained in Wolffe et al.^18^) were significantly more likely to report any mental health condition (OR = 1.5, 1.2–1.7), or anxiety (OR = 1.2, 1.0–1.5) (Fig. 7). Feeling that colleagues uphold this belief was also associated with a slightly increased odds ratio for any mental health condition (OR = 1.2, 1.0–1.3).

The other variables significantly associated with any mental health disorder and anxiety were feeling that cleaning was not taken seriously at the workplace (OR = 1.4, 1.3–1.6 for any mental health condition, and OR = 1.2, 1.0–1.4 for anxiety). In addition, firefighters who had not received training on the health effects of fire contaminants were only slightly (but not significantly) more likely to indicate having any mental health condition (OR = 1.1, 1.0–1.2).

## Discussion

### Occupational exposure to contaminants and mental health disorders in firefighters

UK firefighters were found to have significantly higher rates of (any) self-reported mental health conditions when compared to the general UK population (who were more reliably diagnosed against the UK revised Clinical Interview Schedule (CIS-R)). In particular, the rates of self-reported anxiety and depression in firefighters far exceeded those diagnosed in the general population. The rate of anxiety among surveyed firefighters was twice that of the general population, while the rate of depression was nearly three times that of the general population.

Several measures of potential contaminant exposure were associated with a significantly increased odds ratios for any mental health condition, anxiety, or depression. For example, firefighters noticing soot in the nose/throat for a day or more after washing, were nearly twice as likely to suffer any mental health condition.

Of the potential sources of contaminant exposure assessed in the survey, eating with sooty hands, taking PPE home to clean, wearing poorly fitting PPE, and noticing soot in the nose/throat were most consistently associated with increased odds ratios for mental health disorders, significantly increasing the likelihood of firefighters reporting any mental health condition, anxiety, and (in the case of taking PPE home) depression.

There is very little known about the association between firefighters’ exposures to different contaminants and mental health disorders. Given that firefighters spend prolonged amounts of time attending fire incidents, their capacity for exposure is far greater when compared to the general population and other occupational groups. For example, exposure studies record concentrations of particulate matter (PM) orders of magnitude greater at fire incidents (e.g. wildfires^40^) when compared to ambient concentrations measured in studies of mental health in general populations^41^. This comparison is particularly concerning, given that 84% of surveyed firefighters often/sometimes attend fires without respiratory protective equipment (Wolffe et al.^18^).

Furthermore, recent biomonitoring data has revealed positive associations between urinary concentrations of several polycyclic aromatic hydrocarbons^42^ (a class of chemicals commonly released in fires and found on firefighters’ PPE, workplaces etc.) and mental health disorders (such as depression)^43–45^.

However, most research on the association between mental health disorders and chemicals, particulates etc. found in fire smoke comes from studies focused on identifying genetic risk factors for mental health disorders^46,47^ and exposure to chemicals in early-life (including pre-natal) leading to impeded neurodevelopment^48,49^. There are also epidemiological studies examining the occurrence of mental health disorders in general populations, e.g. associations between common air pollutants (PM_10_ and PM_2.5_, benzene, nitrogen oxides, ozone etc.) and common mental health disorders^41,50^, depression^41,51–53^, and/or anxiety^52^ have been documented^41^.

Further, it is widely recognised that social (e.g. socioeconomic status), psychological (e.g. exposure to trauma), biological (e.g. enduring painful illness), and environmental (e.g. interactions with the physical environment) factors not only vary, but may also have synergistic effects on mental health outcomes. Thus, it is possible that the impact on firefighters may be amplified as firefighters are already occupationally predisposed to several other mental health risk factors (e.g. exposure to trauma/stress etc.).

### Mental health disorders in firefighters and sleeping problems

As well as mental health conditions, poor sleep is known to impact performance, e.g. compromising firefighters’ safety by reducing reaction times^54^. In addition, chronic sleeping problems may also lead to poor immune, cardiovascular, and gastrointestinal health^55^.

Over 60% of surveyed firefighters reported sleeping problems (with shift work pattern being the major reason). By contrast, an estimated 36% of UK adults have sleeping problems^56^. A strong link between sleeping problems and mental health was found among surveyed firefighters (Fig. 4), with firefighters reporting sleeping problems being at least twice as likely to suffer anxiety or depression compared to firefighters without sleeping issues, and over 4 times as likely to report any mental health condition (Fig. 4).

Research suggests that increasing awareness and screening firefighters for sleep disorders as well as revising their shift schedules (in order to minimise disruption to circadian rhythms) may help to maintain healthy sleep schedules^57^.

### Other occupational risk factors for mental health disorders in firefighters

Although the survey did not account for all mental health risk factors, the rate of self-reported PTSD (a condition associated with exposure to trauma) among surveyed firefighters was comparable to that of the UK population (*p* > 0.05). However, as explained earlier, caution needs to be taken when comparing self-reported cases with CIS-R diagnosed cases.

Scientific literature on firefighters’ risk of PTSD is variable in terms of its findings and measures. Some authors have identified being younger, or beginning a career in the Fire and Rescue Service at a young age, as risk factors for developing PTSD^58^. Others have documented a higher prevalence of PTSD among retired when compared to serving firefighters^59^. Thus, given the relatively older modal age of surveyed firefighters (between 45 and 54 years old, Wolffe et al.^18^), and the exclusion of retired/ex-firefighters, it is possible that the prevalence of PTSD in UK firefighters is under-represented in the survey. Similarly, firefighters suffering from PTSD may have been less inclined to participate in the survey.

Other occupational risk factors for mental health disorders among firefighters may include cultural aspects of firefighting, and/or awareness of contaminant exposure and its health effects. Studies in populations who are knowingly exposed to contaminants (e.g. contaminated drinking water etc.) document increased rates of mental health conditions such as anxiety, as a result of concern over the effects of this exposure^60^. Similarly, if firefighters’ awareness of the health effects of contaminant exposure outpaces decontamination policy changes/enforcement, this may cause anxiety among firefighters who feel that their health is not being prioritised.

Similarly, cultural aspects of firefighting (e.g. workplace bravado or the need to remain stoic in high stress or traumatic situations), may negatively impact firefighters’ mental health e.g. by supressing trauma.

## Limitations

Previous parts of the UK Firefighter Contamination Survey analysis already discussed some of the limitations of this survey (Wolffe et al.^18^) such as participation bias. Additionally, the self-reported mental health conditions, studied in this manuscript, were not verified against an official clinical diagnosis.

As the primary focus of the survey was on contaminant control measures, several confounders of mental health were also not accounted for e.g. family history and/or co-occurring diseases which may contribute to the development of mental health disorders^61^.

Quantitation of contaminants in firefighters and/or PPE and workplaces, and how these may change with regards to the contaminant control practices assessed in this survey, would further strengthen suggested relationships between fire contaminant exposure and mental health outcomes.

The analyses conducted in this manuscript found several significant associations between potential contaminant exposure and mental health disorders. However, the manuscript does not assess interaction between these exposure variables. For example, eating while wearing PPE may be associated with eating with sooty hands. Refining a final logistic regression model, in which all relevant exposure variables assessed in the survey are included and interaction accounted for, represents an area for future work. Additionally, a complete case analysis is presented in this manuscript (whereby firefighters who did not answer the question about mental health were excluded from subsequent analyses). Thus, further model development will require imputation methods to account for missing data and reduce potential bias.

It should also be noted that mental health is a topic which continues to be associated with stigma, especially in males^62^. Thus, it is possible that the prevalence of mental health conditions in the survey has been under-represented.

Furthermore, sleeping problems and mental health conditions share mutual causality, whereby mental health conditions can arise from poor sleep, and vice versa. In fact, 46% of firefighters reporting sleeping problems indicated that mental health was the cause. As logistic regression analyses did not distinguish between firefighters who did and did not report mental health as a cause of their sleeping problems, further refinement of logistic regression models should be performed.

Finally, the survey did not ask firefighters about their access to mental health services/support within their workplaces, (a variable known to influence mental health outcomes in firefighters^63^), so cannot give a complete account of issues concerning mental health within the UK Fire and Rescue Service.

## Conclusion

The rate of depression among UK firefighters is nearly three times that of the general population. Additionally, firefighters suffer anxiety at a rate over twice that of the general population, with a slightly higher proportion of firefighters reporting any mental health condition compared to the general population (20% versus 17%). Given the high rates of depression and anxiety, further support for mental health, and focus on mental health interventions, may be required in UK Fire and Rescue Services.

Several health and lifestyle factors were found to be significantly associated with mental health disorders among surveyed firefighters. The most significant among these was sleeping problems. However, adjusting for such risk factors through logistic regression analyses revealed significantly increased anxiety and any mental health condition odds ratios for several measures of contaminant exposure, including noticing soot in the nose/throat, taking PPE home to clean, wearing poorly fitting PPE, and eating with sooty hands. Further analysis on firefighters’ capacity for exposure to fire contaminants is covered in Wolffe et al.^18^.

Analyses conducted in this manuscript can be used to inform more comprehensive predictive models of mental health in the UK FRSs, outlining several contaminant control measures which should be considered in such modelling.

Further research is required in order to better understand the complex relationship between potential contaminant exposure and firefighters’ mental health, including the potentially modifying effects of other risk factors such as psychological, biological, environmental, and genetic factors.

However, given the potentially synergistic effects of these risk factors, it is important to consider interventions which address not only contaminant exposure, but also firefighters’ physical and mental well-being in order to improve the mental health of firefighters in the UK Fire and Rescue Service.

## Supplementary Information

Supplementary Information 1.

Supplementary Information 2.

## Data Availability

The datasets generated and/or analysed during the current study are available from the corresponding author on reasonable request.
