# Supplementary material for: Mental health of UK firefighters
Source: Sci Rep. 2023 Jan 10;13:62. doi: 10.1038/s41598-022-24834-x (PMC9832123; doi:10.1038/s41598-022-24834-x)
Supplement: Supplementary file 2 — Supplementary Information 2. [file 41598_2022_24834_MOESM2_ESM.docx]

**Supplemental File S2**

Mental Health of UK Firefighters

Taylor A. M. Wolffe^1^, Andrew Robinson^1,2^, Anna Clinton^1^, Louis Turrell^1,2^, Anna A. Stec^1,*^

^1^Centre for Fire and Hazards Science, University of Central Lancashire, Preston, Lancashire, PR1 2HE, UK

^2^Royal Preston Hospital, Lancashire Teaching Hospitals NHS Foundation Trust, Preston, Lancashire, PR2 9HT, UK

^*^Corresponding author: [aastec@uclan.ac.uk](mailto:aastec@uclan.ac.uk)

**Additional Survey Methodological Detail**

The survey was piloted with a small subset of firefighters, and questions rephrased for clarity according to feedback. Ethical approval for the survey was granted by the University of Central Lancashire Ethics Committee, and all analyses were conducted in accordance with relevant guidelines and regulations.

The survey ran through Jisc software, for a period of 3 months between November 2019 and February 2020. A link to the survey was distributed to participants via email through the Fire Brigades Union (FBU). The survey took approximately 20 minutes to complete and was supported by UK Fire and Rescue Services (FRSs) with respect to allowing firefighters dedicated time within their workday in which to complete it.

Free text answers were manually coded for analysis according to the most commonly appearing themes.

All currently serving UK firefighters were eligible to take part in the survey. Therefore, the first question of the survey, *“Are you currently working as a firefighter in the UK?”*  was used to include/exclude survey responses from analysis. A total of 6 respondents indicated that they were **not** currently working as firefighters in the UK and were thus excluded from further analysis. A further 4 respondents identified themselves as retired in the free text answers they provided to survey questions and were thus excluded. Four hundred and seventy-one respondents left this initial question blank. Due to the nature of recruitment to the survey (i.e. via email to FBU members), these respondents were assumed to be currently serving UK firefighters and included in subsequent analyses. This left a total of 10,649 included respondents. This figure represents approximately 24% of the UK’s total Firefighter workforce.

**Collinearity diagnostics**

|  | Any Mental Health | | Depression | | Anxiety | | Problems Sleeping | |
| --- | --- | --- | --- | --- | --- | --- | --- | --- |
|  | Tolerance | VIF | Tolerance | VIF | Tolerance | VIF | Tolerance | VIF |
| Any mental health condition | n/a | n/a | n/a | n/a | n/a | n/a | 0.978 | 1.022 |
| Has another mental health condition | n/a | n/a | 0.937 | 1.067 | 0.949 | 1.054 | n/a | n/a |
| Problem_Sleeper | 0.970 | 1.031 | 0.927 | 1.079 | 0.935 | 1.069 | n/a | n/a |
| Excessive_drinker | 0.957 | 1.045 | 0.956 | 1.046 | 0.956 | 1.046 | 0.957 | 1.044 |
| Smoker | 0.980 | 1.020 | 0.978 | 1.022 | 0.978 | 1.023 | 0.980 | 1.021 |
| Exercise_infrequently | 0.967 | 1.035 | 0.967 | 1.035 | 0.966 | 1.035 | 0.966 | 1.035 |
| Managerial_Role | 0.857 | 1.166 | 0.857 | 1.167 | 0.857 | 1.166 | 0.857 | 1.166 |
| Diabetes | 0.991 | 1.009 | 0.991 | 1.009 | 0.991 | 1.009 | 0.991 | 1.009 |
| BP_Problems | 0.944 | 1.059 | 0.940 | 1.063 | 0.942 | 1.062 | 0.942 | 1.061 |
| Cancer | 0.982 | 1.018 | 0.981 | 1.019 | 0.982 | 1.018 | 0.981 | 1.019 |
| Fertility_Problems | 0.993 | 1.007 | 0.992 | 1.008 | 0.992 | 1.008 | 0.992 | 1.008 |
| Attend_fires_weekly | 0.971 | 1.030 | 0.971 | 1.030 | 0.971 | 1.030 | 0.973 | 1.028 |
| Age_pseudo_contin | 0.375 | 2.670 | 0.374 | 2.672 | 0.374 | 2.671 | 0.376 | 2.660 |
| Length_Service_pseudo_contin | 0.346 | 2.894 | 0.345 | 2.900 | 0.345 | 2.898 | 0.345 | 2.899 |

**Table S1: Co-linearity statistics for variables considered in logistic regression models.**

**Final Logistic Regression Models: Mental Health Conditions**

| Mental Health Condition | Details | Variables included in logistic regression model | Variables excluded in logistic regression | Hosmer and Lemeshow Goodness of Fit | Model Classification |
| --- | --- | --- | --- | --- | --- |
| Any | Firefighters with at least one mental health condition were compared to those who indicated that they had no known health conditions | - - - - Problems sleeping       - Excessive drinking       - Smoking       - Exercising infrequently       - Managerial role       - Blood pressure problems       - Cancer diagnosis       - Fertility problems       - Length of service | - Attending fires on a weekly basis - Diabetes - Age | 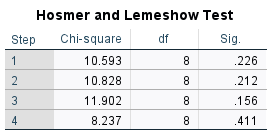 | 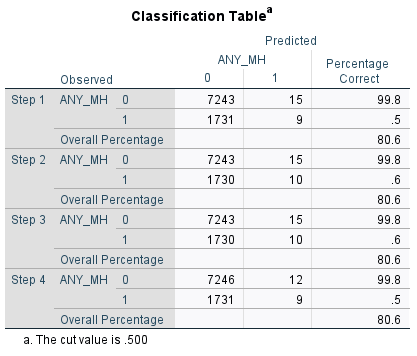 |
| Depression | Firefighters who indicated that they had depression were compared to those who did not have depression (even if those with/without depression had other mental health conditions) | - Having another mental condition - Problems sleeping - Exercising infrequently - Attending fires on a weekly basis | - Excessive drinking - Smoking - Managerial role - Diabetes - Blood pressure problems - Cancer diagnosis - Fertility problems - Length of service - Age | 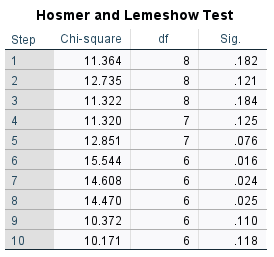 | 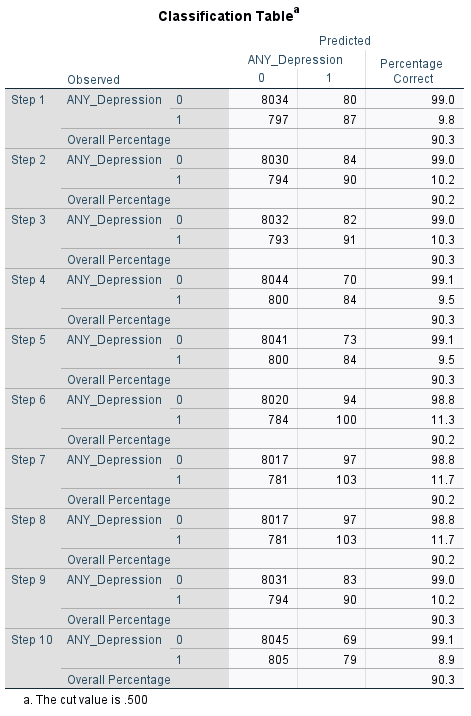 |
| Anxiety | Firefighters who indicated that they had anxiety were compared to those who did not have anxiety (even if those with/without anxiety had other mental health conditions) | - Having another mental health condition - Problems sleeping - Managerial role - Blood pressure problems - Fertility problems | - Excessive drinking - Smoking - Exercising infrequently - Diabetes - Cancer diagnosis - Attending fires on a weekly basis - Length of Service - Age | 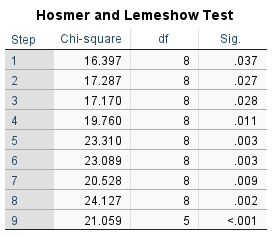 | 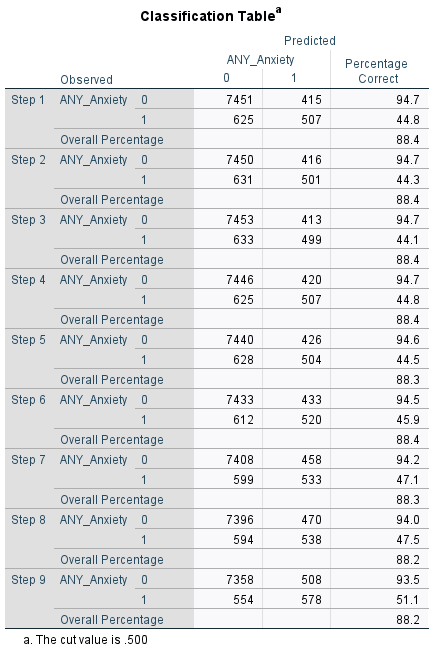 |
| Problems Sleeping | Firefighters who indicated problems sleeping compared to those who did not indicate problems sleeping | - Any mental health condition - Excessive drinking - Smoking - Blood pressure problems - Fertility problems - Attending fires on at least a weekly basis - Age | - Exercising infrequently - Managerial Role - Diabetes - Cancer diagnosis - Length of service | 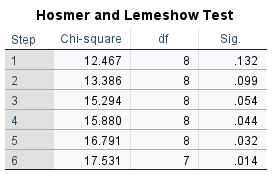 | 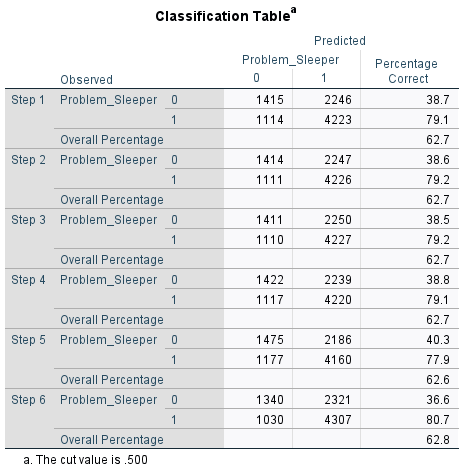 |

**Table S2: Final logistic regression models for analysis of associations between potential contaminant exposure and any mental health condition, depression, or anxiety in UK Firefighters. Note that a step-wise backward selection method was used to arrive at the final models.**

**Logistic Regression Model Refinement**

**Any Mental Health Condition**


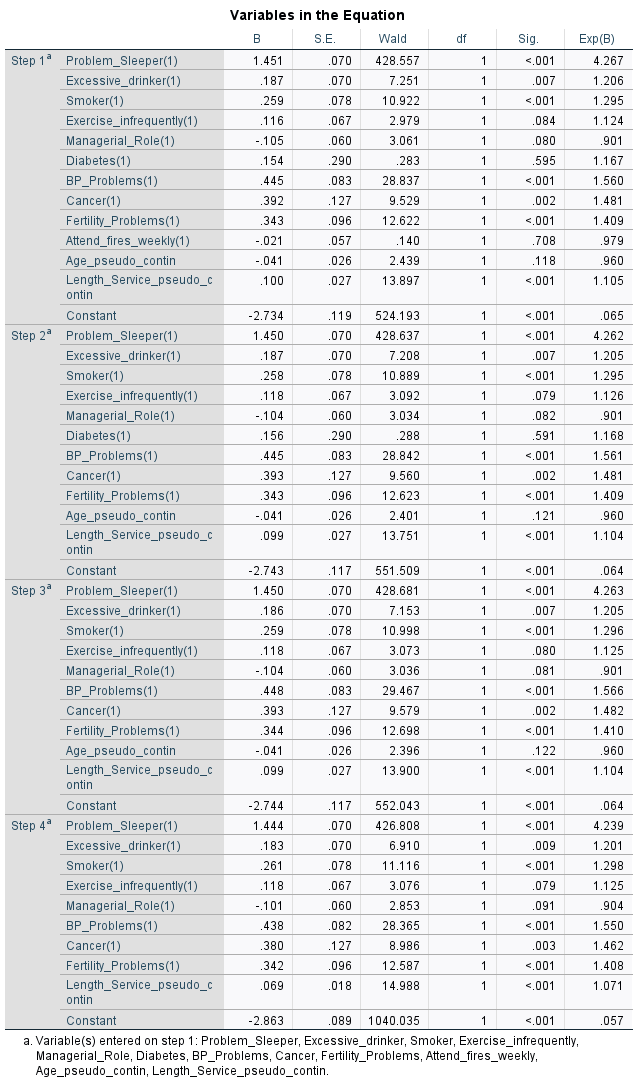


**Table S3: Backward stepwise selection for any mental health condition model refinement.**

**Depression**

| **Variables in the Equation** | | | | | | | |
| --- | --- | --- | --- | --- | --- | --- | --- |
|  | | B | S.E. | Wald | df | Sig. | Exp(B) |
| Step 1^a^ | Has_another_MH(1) | 2.733 | .084 | 1055.240 | 1 | <.001 | 15.374 |
|  | Problem_Sleeper(1) | .813 | .106 | 58.916 | 1 | <.001 | 2.254 |
|  | Excessive_drinker(1) | .070 | .101 | .489 | 1 | .484 | 1.073 |
|  | Smoker(1) | .106 | .112 | .898 | 1 | .343 | 1.112 |
|  | Exercise_infrequently(1) | .312 | .096 | 10.593 | 1 | .001 | 1.366 |
|  | Managerial_Role(1) | .010 | .087 | .012 | 1 | .911 | 1.010 |
|  | Diabetes(1) | -.234 | .431 | .296 | 1 | .586 | .791 |
|  | BP_Problems(1) | .166 | .119 | 1.968 | 1 | .161 | 1.181 |
|  | Cancer(1) | -.159 | .192 | .688 | 1 | .407 | .853 |
|  | Fertility_Problems(1) | .179 | .139 | 1.659 | 1 | .198 | 1.196 |
|  | Attend_fires_weekly(1) | .155 | .083 | 3.481 | 1 | .062 | 1.168 |
|  | Age_pseudo_contin | .013 | .039 | .112 | 1 | .738 | 1.013 |
|  | Length_Service_pseudo_contin | -.006 | .040 | .022 | 1 | .882 | .994 |
|  | Constant | -4.074 | .180 | 510.557 | 1 | <.001 | .017 |
| Step 2^a^ | Has_another_MH(1) | 2.732 | .084 | 1056.044 | 1 | <.001 | 15.369 |
|  | Problem_Sleeper(1) | .813 | .106 | 58.932 | 1 | <.001 | 2.254 |
|  | Excessive_drinker(1) | .070 | .101 | .490 | 1 | .484 | 1.073 |
|  | Smoker(1) | .106 | .112 | .894 | 1 | .344 | 1.112 |
|  | Exercise_infrequently(1) | .312 | .095 | 10.721 | 1 | .001 | 1.367 |
|  | Diabetes(1) | -.235 | .431 | .298 | 1 | .585 | .790 |
|  | BP_Problems(1) | .166 | .119 | 1.966 | 1 | .161 | 1.181 |
|  | Cancer(1) | -.159 | .192 | .687 | 1 | .407 | .853 |
|  | Fertility_Problems(1) | .179 | .138 | 1.679 | 1 | .195 | 1.196 |
|  | Attend_fires_weekly(1) | .155 | .083 | 3.470 | 1 | .062 | 1.168 |
|  | Age_pseudo_contin | .013 | .039 | .109 | 1 | .742 | 1.013 |
|  | Length_Service_pseudo_contin | -.005 | .038 | .016 | 1 | .901 | .995 |
|  | Constant | -4.072 | .180 | 512.364 | 1 | <.001 | .017 |
| Step 3^a^ | Has_another_MH(1) | 2.732 | .084 | 1058.963 | 1 | <.001 | 15.360 |
|  | Problem_Sleeper(1) | .813 | .106 | 58.939 | 1 | <.001 | 2.254 |
|  | Excessive_drinker(1) | .070 | .100 | .480 | 1 | .488 | 1.072 |
|  | Smoker(1) | .106 | .112 | .908 | 1 | .341 | 1.112 |
|  | Exercise_infrequently(1) | .312 | .095 | 10.715 | 1 | .001 | 1.366 |
|  | Diabetes(1) | -.237 | .430 | .303 | 1 | .582 | .789 |
|  | BP_Problems(1) | .165 | .118 | 1.951 | 1 | .163 | 1.180 |
|  | Cancer(1) | -.160 | .192 | .692 | 1 | .406 | .853 |
|  | Fertility_Problems(1) | .180 | .138 | 1.681 | 1 | .195 | 1.197 |
|  | Attend_fires_weekly(1) | .154 | .083 | 3.464 | 1 | .063 | 1.166 |
|  | Age_pseudo_contin | .009 | .025 | .129 | 1 | .720 | 1.009 |
|  | Constant | -4.068 | .176 | 531.472 | 1 | <.001 | .017 |
| Step 4^a^ | Has_another_MH(1) | 2.732 | .084 | 1058.989 | 1 | <.001 | 15.361 |
|  | Problem_Sleeper(1) | .816 | .106 | 59.786 | 1 | <.001 | 2.261 |
|  | Excessive_drinker(1) | .075 | .099 | .568 | 1 | .451 | 1.078 |
|  | Smoker(1) | .104 | .111 | .863 | 1 | .353 | 1.109 |
|  | Exercise_infrequently(1) | .313 | .095 | 10.845 | 1 | <.001 | 1.368 |
|  | Diabetes(1) | -.227 | .429 | .280 | 1 | .597 | .797 |
|  | BP_Problems(1) | .173 | .116 | 2.223 | 1 | .136 | 1.189 |
|  | Cancer(1) | -.152 | .191 | .636 | 1 | .425 | .859 |
|  | Fertility_Problems(1) | .179 | .138 | 1.675 | 1 | .196 | 1.196 |
|  | Attend_fires_weekly(1) | .156 | .082 | 3.567 | 1 | .059 | 1.168 |
|  | Constant | -4.020 | .113 | 1268.186 | 1 | <.001 | .018 |
| Step 5^a^ | Has_another_MH(1) | 2.731 | .084 | 1058.760 | 1 | <.001 | 15.353 |
|  | Problem_Sleeper(1) | .815 | .105 | 59.688 | 1 | <.001 | 2.259 |
|  | Excessive_drinker(1) | .074 | .099 | .557 | 1 | .455 | 1.077 |
|  | Smoker(1) | .103 | .111 | .849 | 1 | .357 | 1.108 |
|  | Exercise_infrequently(1) | .314 | .095 | 10.917 | 1 | <.001 | 1.369 |
|  | BP_Problems(1) | .169 | .116 | 2.124 | 1 | .145 | 1.184 |
|  | Cancer(1) | -.156 | .191 | .667 | 1 | .414 | .856 |
|  | Fertility_Problems(1) | .179 | .138 | 1.676 | 1 | .195 | 1.196 |
|  | Attend_fires_weekly(1) | .156 | .082 | 3.587 | 1 | .058 | 1.169 |
|  | Constant | -4.020 | .113 | 1268.933 | 1 | <.001 | .018 |
| Step 6^a^ | Has_another_MH(1) | 2.734 | .084 | 1062.900 | 1 | <.001 | 15.400 |
|  | Problem_Sleeper(1) | .817 | .105 | 60.062 | 1 | <.001 | 2.264 |
|  | Smoker(1) | .108 | .111 | .945 | 1 | .331 | 1.114 |
|  | Exercise_infrequently(1) | .318 | .095 | 11.256 | 1 | <.001 | 1.375 |
|  | BP_Problems(1) | .173 | .116 | 2.239 | 1 | .135 | 1.189 |
|  | Cancer(1) | -.156 | .191 | .665 | 1 | .415 | .856 |
|  | Fertility_Problems(1) | .179 | .138 | 1.672 | 1 | .196 | 1.196 |
|  | Attend_fires_weekly(1) | .160 | .082 | 3.756 | 1 | .053 | 1.173 |
|  | Constant | -4.012 | .112 | 1276.106 | 1 | <.001 | .018 |
| Step 7^a^ | Has_another_MH(1) | 2.731 | .084 | 1063.477 | 1 | <.001 | 15.348 |
|  | Problem_Sleeper(1) | .817 | .105 | 60.009 | 1 | <.001 | 2.263 |
|  | Smoker(1) | .109 | .111 | .956 | 1 | .328 | 1.115 |
|  | Exercise_infrequently(1) | .319 | .095 | 11.302 | 1 | <.001 | 1.376 |
|  | BP_Problems(1) | .169 | .116 | 2.149 | 1 | .143 | 1.185 |
|  | Fertility_Problems(1) | .174 | .138 | 1.577 | 1 | .209 | 1.190 |
|  | Attend_fires_weekly(1) | .160 | .082 | 3.761 | 1 | .052 | 1.173 |
|  | Constant | -4.017 | .112 | 1281.530 | 1 | <.001 | .018 |
| Step 8^a^ | Has_another_MH(1) | 2.735 | .084 | 1068.710 | 1 | <.001 | 15.408 |
|  | Problem_Sleeper(1) | .819 | .105 | 60.439 | 1 | <.001 | 2.269 |
|  | Exercise_infrequently(1) | .326 | .095 | 11.872 | 1 | <.001 | 1.385 |
|  | BP_Problems(1) | .168 | .116 | 2.119 | 1 | .145 | 1.183 |
|  | Fertility_Problems(1) | .173 | .138 | 1.570 | 1 | .210 | 1.189 |
|  | Attend_fires_weekly(1) | .161 | .082 | 3.821 | 1 | .051 | 1.174 |
|  | Constant | -4.007 | .112 | 1287.665 | 1 | <.001 | .018 |
| Step 9^a^ | Has_another_MH(1) | 2.738 | .084 | 1072.832 | 1 | <.001 | 15.463 |
|  | Problem_Sleeper(1) | .824 | .105 | 61.104 | 1 | <.001 | 2.279 |
|  | Exercise_infrequently(1) | .329 | .095 | 12.103 | 1 | <.001 | 1.389 |
|  | BP_Problems(1) | .171 | .115 | 2.191 | 1 | .139 | 1.186 |
|  | Attend_fires_weekly(1) | .160 | .082 | 3.797 | 1 | .051 | 1.174 |
|  | Constant | -3.997 | .111 | 1289.221 | 1 | <.001 | .018 |
| Step 10^a^ | Has_another_MH(1) | 2.747 | .083 | 1085.275 | 1 | <.001 | 15.604 |
|  | Problem_Sleeper(1) | .833 | .105 | 62.858 | 1 | <.001 | 2.301 |
|  | Exercise_infrequently(1) | .337 | .094 | 12.723 | 1 | <.001 | 1.400 |
|  | Attend_fires_weekly(1) | .164 | .082 | 3.977 | 1 | .046 | 1.178 |
|  | Constant | -3.990 | .111 | 1286.951 | 1 | <.001 | .019 |
| a. Variable(s) entered on step 1: Has_another_MH, Problem_Sleeper, Excessive_drinker, Smoker, Exercise_infrequently, Managerial_Role, Diabetes, BP_Problems, Cancer, Fertility_Problems, Attend_fires_weekly, Age_pseudo_contin, Length_Service_pseudo_contin. | | | | | | | |

**Table S5: Backward stepwise selection for depression model refinement.**

**Anxiety**

| **Variables in the Equation** | | | | | | | |
| --- | --- | --- | --- | --- | --- | --- | --- |
|  | | B | S.E. | Wald | df | Sig. | Exp(B) |
| Step 1^a^ | Has_another_MH(1) | 2.576 | .075 | 1168.220 | 1 | <.001 | 13.147 |
|  | Problem_Sleeper(1) | 1.049 | .094 | 125.562 | 1 | <.001 | 2.854 |
|  | Excessive_drinker(1) | .097 | .093 | 1.092 | 1 | .296 | 1.101 |
|  | Smoker(1) | .037 | .104 | .125 | 1 | .724 | 1.037 |
|  | Exercise_infrequently(1) | .106 | .089 | 1.427 | 1 | .232 | 1.112 |
|  | Managerial_Role(1) | -.255 | .080 | 10.126 | 1 | .001 | .775 |
|  | Diabetes(1) | .074 | .381 | .037 | 1 | .847 | 1.076 |
|  | BP_Problems(1) | .294 | .110 | 7.151 | 1 | .007 | 1.342 |
|  | Cancer(1) | .219 | .170 | 1.658 | 1 | .198 | 1.245 |
|  | Fertility_Problems(1) | .266 | .127 | 4.362 | 1 | .037 | 1.305 |
|  | Attend_fires_weekly(1) | .012 | .075 | .025 | 1 | .874 | 1.012 |
|  | Age_pseudo_contin | -.053 | .035 | 2.296 | 1 | .130 | .949 |
|  | Length_Service_pseudo_contin | .063 | .036 | 3.092 | 1 | .079 | 1.065 |
|  | Constant | -3.376 | .157 | 459.597 | 1 | <.001 | .034 |
| Step 2^a^ | Has_another_MH(1) | 2.576 | .075 | 1168.228 | 1 | <.001 | 13.146 |
|  | Problem_Sleeper(1) | 1.049 | .094 | 125.905 | 1 | <.001 | 2.855 |
|  | Excessive_drinker(1) | .097 | .092 | 1.101 | 1 | .294 | 1.102 |
|  | Smoker(1) | .037 | .104 | .127 | 1 | .722 | 1.038 |
|  | Exercise_infrequently(1) | .105 | .089 | 1.408 | 1 | .235 | 1.111 |
|  | Managerial_Role(1) | -.255 | .080 | 10.161 | 1 | .001 | .775 |
|  | Diabetes(1) | .073 | .381 | .036 | 1 | .849 | 1.075 |
|  | BP_Problems(1) | .294 | .110 | 7.147 | 1 | .008 | 1.342 |
|  | Cancer(1) | .219 | .170 | 1.653 | 1 | .199 | 1.245 |
|  | Fertility_Problems(1) | .266 | .127 | 4.354 | 1 | .037 | 1.305 |
|  | Age_pseudo_contin | -.053 | .035 | 2.325 | 1 | .127 | .948 |
|  | Length_Service_pseudo_contin | .063 | .035 | 3.209 | 1 | .073 | 1.065 |
|  | Constant | -3.370 | .154 | 480.983 | 1 | <.001 | .034 |
| Step 3^a^ | Has_another_MH(1) | 2.576 | .075 | 1168.277 | 1 | <.001 | 13.147 |
|  | Problem_Sleeper(1) | 1.049 | .094 | 125.919 | 1 | <.001 | 2.855 |
|  | Excessive_drinker(1) | .097 | .092 | 1.097 | 1 | .295 | 1.102 |
|  | Smoker(1) | .037 | .104 | .129 | 1 | .719 | 1.038 |
|  | Exercise_infrequently(1) | .105 | .089 | 1.403 | 1 | .236 | 1.111 |
|  | Managerial_Role(1) | -.255 | .080 | 10.158 | 1 | .001 | .775 |
|  | BP_Problems(1) | .296 | .110 | 7.242 | 1 | .007 | 1.344 |
|  | Cancer(1) | .219 | .170 | 1.658 | 1 | .198 | 1.245 |
|  | Fertility_Problems(1) | .266 | .127 | 4.361 | 1 | .037 | 1.305 |
|  | Age_pseudo_contin | -.053 | .035 | 2.319 | 1 | .128 | .948 |
|  | Length_Service_pseudo_contin | .063 | .035 | 3.237 | 1 | .072 | 1.066 |
|  | Constant | -3.371 | .154 | 481.640 | 1 | <.001 | .034 |
| Step 4^a^ | Has_another_MH(1) | 2.578 | .075 | 1173.883 | 1 | <.001 | 13.169 |
|  | Problem_Sleeper(1) | 1.050 | .093 | 126.370 | 1 | <.001 | 2.859 |
|  | Excessive_drinker(1) | .099 | .092 | 1.160 | 1 | .281 | 1.104 |
|  | Exercise_infrequently(1) | .108 | .088 | 1.485 | 1 | .223 | 1.114 |
|  | Managerial_Role(1) | -.256 | .080 | 10.220 | 1 | .001 | .774 |
|  | BP_Problems(1) | .296 | .110 | 7.247 | 1 | .007 | 1.344 |
|  | Cancer(1) | .220 | .170 | 1.663 | 1 | .197 | 1.246 |
|  | Fertility_Problems(1) | .265 | .127 | 4.338 | 1 | .037 | 1.304 |
|  | Age_pseudo_contin | -.053 | .035 | 2.343 | 1 | .126 | .948 |
|  | Length_Service_pseudo_contin | .063 | .035 | 3.195 | 1 | .074 | 1.065 |
|  | Constant | -3.365 | .153 | 486.673 | 1 | <.001 | .035 |
| Step 5^a^ | Has_another_MH(1) | 2.581 | .075 | 1177.777 | 1 | <.001 | 13.207 |
|  | Problem_Sleeper(1) | 1.054 | .093 | 127.325 | 1 | <.001 | 2.869 |
|  | Exercise_infrequently(1) | .112 | .088 | 1.613 | 1 | .204 | 1.119 |
|  | Managerial_Role(1) | -.256 | .080 | 10.274 | 1 | .001 | .774 |
|  | BP_Problems(1) | .298 | .110 | 7.382 | 1 | .007 | 1.348 |
|  | Cancer(1) | .215 | .170 | 1.596 | 1 | .207 | 1.240 |
|  | Fertility_Problems(1) | .266 | .127 | 4.366 | 1 | .037 | 1.305 |
|  | Age_pseudo_contin | -.052 | .035 | 2.220 | 1 | .136 | .950 |
|  | Length_Service_pseudo_contin | .066 | .035 | 3.494 | 1 | .062 | 1.068 |
|  | Constant | -3.369 | .152 | 488.251 | 1 | <.001 | .034 |
| Step 6^a^ | Has_another_MH(1) | 2.582 | .075 | 1179.588 | 1 | <.001 | 13.228 |
|  | Problem_Sleeper(1) | 1.054 | .093 | 127.383 | 1 | <.001 | 2.869 |
|  | Exercise_infrequently(1) | .110 | .088 | 1.560 | 1 | .212 | 1.116 |
|  | Managerial_Role(1) | -.256 | .080 | 10.251 | 1 | .001 | .774 |
|  | BP_Problems(1) | .300 | .110 | 7.472 | 1 | .006 | 1.350 |
|  | Fertility_Problems(1) | .275 | .127 | 4.666 | 1 | .031 | 1.316 |
|  | Age_pseudo_contin | -.049 | .035 | 1.998 | 1 | .158 | .952 |
|  | Length_Service_pseudo_contin | .066 | .035 | 3.537 | 1 | .060 | 1.068 |
|  | Constant | -3.378 | .152 | 491.537 | 1 | <.001 | .034 |
| Step 7^a^ | Has_another_MH(1) | 2.585 | .075 | 1182.305 | 1 | <.001 | 13.260 |
|  | Problem_Sleeper(1) | 1.054 | .093 | 127.526 | 1 | <.001 | 2.870 |
|  | Managerial_Role(1) | -.248 | .080 | 9.683 | 1 | .002 | .780 |
|  | BP_Problems(1) | .306 | .110 | 7.784 | 1 | .005 | 1.358 |
|  | Fertility_Problems(1) | .277 | .127 | 4.762 | 1 | .029 | 1.320 |
|  | Age_pseudo_contin | -.049 | .035 | 2.027 | 1 | .154 | .952 |
|  | Length_Service_pseudo_contin | .068 | .035 | 3.704 | 1 | .054 | 1.070 |
|  | Constant | -3.363 | .152 | 491.170 | 1 | <.001 | .035 |
| Step 8^a^ | Has_another_MH(1) | 2.587 | .075 | 1184.680 | 1 | <.001 | 13.283 |
|  | Problem_Sleeper(1) | 1.047 | .093 | 126.264 | 1 | <.001 | 2.850 |
|  | Managerial_Role(1) | -.242 | .080 | 9.263 | 1 | .002 | .785 |
|  | BP_Problems(1) | .294 | .109 | 7.250 | 1 | .007 | 1.342 |
|  | Fertility_Problems(1) | .274 | .127 | 4.638 | 1 | .031 | 1.315 |
|  | Length_Service_pseudo_contin | .030 | .023 | 1.684 | 1 | .194 | 1.030 |
|  | Constant | -3.507 | .114 | 938.076 | 1 | <.001 | .030 |
| Step 9^a^ | Has_another_MH(1) | 2.591 | .075 | 1190.444 | 1 | <.001 | 13.340 |
|  | Problem_Sleeper(1) | 1.056 | .093 | 129.108 | 1 | <.001 | 2.876 |
|  | Managerial_Role(1) | -.208 | .075 | 7.668 | 1 | .006 | .812 |
|  | BP_Problems(1) | .322 | .107 | 9.014 | 1 | .003 | 1.380 |
|  | Fertility_Problems(1) | .272 | .127 | 4.562 | 1 | .033 | 1.312 |
|  | Constant | -3.416 | .090 | 1453.502 | 1 | .000 | .033 |
| a. Variable(s) entered on step 1: Has_another_MH, Problem_Sleeper, Excessive_drinker, Smoker, Exercise_infrequently, Managerial_Role, Diabetes, BP_Problems, Cancer, Fertility_Problems, Attend_fires_weekly, Age_pseudo_contin, Length_Service_pseudo_contin. | | | | | | | |

**Table S6: Backward stepwise selection for anxiety model refinement.**

**Logistic Regression Model: Problems Sleeping**

| **Variables in the Equation** | | | | | | | |
| --- | --- | --- | --- | --- | --- | --- | --- |
|  | | B | S.E. | Wald | df | Sig. | Exp(B) |
| Step 1^a^ | ANY_MH(1) | 1.447 | .070 | 426.392 | 1 | <.001 | 4.251 |
|  | Excessive_drinker(1) | .193 | .062 | 9.798 | 1 | .002 | 1.213 |
|  | Smoker(1) | .208 | .069 | 9.058 | 1 | .003 | 1.231 |
|  | Exercise_infrequently(1) | .071 | .057 | 1.551 | 1 | .213 | 1.074 |
|  | Managerial_Role(1) | .068 | .049 | 1.917 | 1 | .166 | 1.071 |
|  | Diabetes(1) | .050 | .276 | .032 | 1 | .857 | 1.051 |
|  | BP_Problems(1) | .366 | .082 | 19.926 | 1 | <.001 | 1.442 |
|  | Cancer(1) | .039 | .120 | .103 | 1 | .748 | 1.039 |
|  | Fertility_Problems(1) | .223 | .089 | 6.276 | 1 | .012 | 1.250 |
|  | Attend_fires_weekly(1) | .196 | .046 | 18.618 | 1 | <.001 | 1.217 |
|  | Age_pseudo_contin | .120 | .021 | 33.130 | 1 | <.001 | 1.128 |
|  | Length_Service_pseudo_contin | -.005 | .022 | .063 | 1 | .801 | .995 |
|  | Constant | -.791 | .084 | 89.079 | 1 | <.001 | .453 |
| Step 2^a^ | ANY_MH(1) | 1.447 | .070 | 426.444 | 1 | <.001 | 4.251 |
|  | Excessive_drinker(1) | .193 | .062 | 9.790 | 1 | .002 | 1.213 |
|  | Smoker(1) | .208 | .069 | 9.077 | 1 | .003 | 1.231 |
|  | Exercise_infrequently(1) | .071 | .057 | 1.549 | 1 | .213 | 1.074 |
|  | Managerial_Role(1) | .068 | .049 | 1.919 | 1 | .166 | 1.071 |
|  | BP_Problems(1) | .367 | .082 | 20.115 | 1 | <.001 | 1.443 |
|  | Cancer(1) | .039 | .120 | .103 | 1 | .749 | 1.039 |
|  | Fertility_Problems(1) | .223 | .089 | 6.289 | 1 | .012 | 1.250 |
|  | Attend_fires_weekly(1) | .196 | .046 | 18.612 | 1 | <.001 | 1.217 |
|  | Age_pseudo_contin | .120 | .021 | 33.115 | 1 | <.001 | 1.128 |
|  | Length_Service_pseudo_contin | -.005 | .022 | .061 | 1 | .805 | .995 |
|  | Constant | -.791 | .084 | 89.084 | 1 | <.001 | .453 |
| Step 3^a^ | ANY_MH(1) | 1.447 | .070 | 426.571 | 1 | <.001 | 4.249 |
|  | Excessive_drinker(1) | .192 | .061 | 9.730 | 1 | .002 | 1.211 |
|  | Smoker(1) | .208 | .069 | 9.139 | 1 | .003 | 1.232 |
|  | Exercise_infrequently(1) | .070 | .057 | 1.525 | 1 | .217 | 1.073 |
|  | Managerial_Role(1) | .065 | .048 | 1.872 | 1 | .171 | 1.067 |
|  | BP_Problems(1) | .366 | .082 | 20.058 | 1 | <.001 | 1.442 |
|  | Cancer(1) | .038 | .120 | .099 | 1 | .753 | 1.039 |
|  | Fertility_Problems(1) | .223 | .089 | 6.294 | 1 | .012 | 1.250 |
|  | Attend_fires_weekly(1) | .195 | .045 | 18.624 | 1 | <.001 | 1.215 |
|  | Age_pseudo_contin | .116 | .014 | 71.563 | 1 | <.001 | 1.123 |
|  | Constant | -.787 | .081 | 93.226 | 1 | <.001 | .455 |
| Step 4^a^ | ANY_MH(1) | 1.447 | .070 | 427.337 | 1 | <.001 | 4.252 |
|  | Excessive_drinker(1) | .192 | .061 | 9.735 | 1 | .002 | 1.211 |
|  | Smoker(1) | .208 | .069 | 9.125 | 1 | .003 | 1.232 |
|  | Exercise_infrequently(1) | .070 | .057 | 1.512 | 1 | .219 | 1.073 |
|  | Managerial_Role(1) | .065 | .048 | 1.870 | 1 | .171 | 1.067 |
|  | BP_Problems(1) | .366 | .082 | 20.087 | 1 | <.001 | 1.443 |
|  | Fertility_Problems(1) | .224 | .089 | 6.364 | 1 | .012 | 1.251 |
|  | Attend_fires_weekly(1) | .195 | .045 | 18.630 | 1 | <.001 | 1.215 |
|  | Age_pseudo_contin | .117 | .014 | 73.004 | 1 | <.001 | 1.124 |
|  | Constant | -.788 | .081 | 93.861 | 1 | <.001 | .455 |
| Step 5^a^ | ANY_MH(1) | 1.449 | .070 | 428.497 | 1 | <.001 | 4.259 |
|  | Excessive_drinker(1) | .192 | .061 | 9.772 | 1 | .002 | 1.212 |
|  | Smoker(1) | .215 | .069 | 9.816 | 1 | .002 | 1.240 |
|  | Managerial_Role(1) | .071 | .047 | 2.269 | 1 | .132 | 1.074 |
|  | BP_Problems(1) | .371 | .082 | 20.596 | 1 | <.001 | 1.449 |
|  | Fertility_Problems(1) | .225 | .089 | 6.431 | 1 | .011 | 1.253 |
|  | Attend_fires_weekly(1) | .192 | .045 | 18.043 | 1 | <.001 | 1.211 |
|  | Age_pseudo_contin | .117 | .014 | 74.270 | 1 | <.001 | 1.125 |
|  | Constant | -.781 | .081 | 92.708 | 1 | <.001 | .458 |
| Step 6^a^ | ANY_MH(1) | 1.449 | .070 | 428.316 | 1 | <.001 | 4.257 |
|  | Excessive_drinker(1) | .194 | .061 | 10.024 | 1 | .002 | 1.215 |
|  | Smoker(1) | .213 | .069 | 9.641 | 1 | .002 | 1.238 |
|  | BP_Problems(1) | .374 | .082 | 21.032 | 1 | <.001 | 1.454 |
|  | Fertility_Problems(1) | .230 | .089 | 6.713 | 1 | .010 | 1.259 |
|  | Attend_fires_weekly(1) | .193 | .045 | 18.264 | 1 | <.001 | 1.213 |
|  | Age_pseudo_contin | .123 | .013 | 86.282 | 1 | <.001 | 1.130 |
|  | Constant | -.783 | .081 | 93.280 | 1 | <.001 | .457 |
| a. Variable(s) entered on step 1: ANY_MH, Excessive_drinker, Smoker, Exercise_infrequently, Managerial_Role, Diabetes, BP_Problems, Cancer, Fertility_Problems, Attend_fires_weekly, Age_pseudo_contin, Length_Service_pseudo_contin. | | | | | | | |

**Table S7:** **Backward stepwise selection for problems sleeping model refinement.**ANY_MH refers to firefighters suffering at least one mental health condition.
